# Bridging the Antimicrobial Activity of Two Lactoferricin Derivatives in *E. coli* and Lipid-Only Membranes

**DOI:** 10.3389/fmedt.2021.625975

**Published:** 2021-02-24

**Authors:** Lisa Marx, Enrico F. Semeraro, Johannes Mandl, Johannes Kremser, Moritz P. Frewein, Nermina Malanovic, Karl Lohner, Georg Pabst

**Affiliations:** ^1^Department of Biophysics, Institute of Molecular Biosciences, University of Graz, Graz, Austria; ^2^BioTechMed Graz, Graz, Austria; ^3^Field of Excellence BioHealth—University of Graz, Graz, Austria; ^4^Soft Matter Science and Support Group, Institut Laue-Langevin, Grenoble, France

**Keywords:** antimicrobial peptides, lactoferricin, minimum inhibitory concentration, partitioning, zeta-potential, dye-leakage assay, tryptophan fluorescence, small-angle X-ray scattering

## Abstract

We coupled the antimicrobial activity of two well-studied lactoferricin derivatives, LF11-215 and LF11-324, in *Escherichia coli* and different lipid-only mimics of its cytoplasmic membrane using a common thermodynamic framework for peptide partitioning. In particular, we combined an improved analysis of microdilution assays with ζ-potential measurements, which allowed us to discriminate between the maximum number of surface-adsorbed peptides and peptides fully partitioned into the bacteria. At the same time, we measured the partitioning of the peptides into vesicles composed of phosphatidylethanolamine (PE), phosphatidylgylcerol (PG), and cardiolipin (CL) mixtures using tryptophan fluorescence and determined their membrane activity using a dye leakage assay and small-angle X-ray scattering. We found that the vast majority of LF11-215 and LF11-324 readily enter inner bacterial compartments, whereas only 1−5% remain surface bound. We observed comparable membrane binding of both peptides in membrane mimics containing PE and different molar ratios of PG and CL. The peptides' activity caused a concentration-dependent dye leakage in all studied membrane mimics; however, it also led to the formation of large aggregates, part of which contained collapsed multibilayers with sandwiched peptides in the interstitial space between membranes. This effect was least pronounced in pure PG vesicles, requiring also the highest peptide concentration to induce membrane permeabilization. In PE-containing systems, we additionally observed an effective shielding of the fluorescent dyes from leakage even at highest peptide concentrations, suggesting a coupling of the peptide activity to vesicle fusion, being mediated by the intrinsic lipid curvatures of PE and CL. Our results thus show that LF11-215 and LF11-324 effectively target inner bacterial components, while the stored elastic stress makes membranes more vulnerable to peptide translocation.

## 1. Introduction

The history of research on antimicrobial peptides (AMPs) as promising agents to combat infectious diseases is long and rich in diverse aspects. Studied for about four decades (1), AMPs continue to spur significant research efforts by their ability to discriminate the lipid architecture of cell envelopes, and evade classical resistance mechanisms based on direct molecular (key-lock) interactions, which increasingly limits medical treatments by conventional antibiotics [for review, see e.g., (2, 3)].

AMPs are typically composed of cationic and hydrophobic amino acids making them highly membrane active. Responses of their target membranes depend on the AMPs' physicochemical properties (primary structure, hydrophobic moment, length, etc.) and concentration (partitioning), as revealed from studies on membrane model systems, and include membrane thinning (4), or thickening (5), lipid flip/flop (6) or changes in membrane elasticity (7) at relatively low concentrations. Pore-formation (barrel-stave, toroidal/worm-hole) (8) or changes in membrane topology (9, 10) have been reported at elevated AMP concentrations, eventually leading to complete micellization (11) of the membrane, also known as the carpet model (12). It is increasingly becoming clear, however, that the observed effects strongly depend on the lipid composition of the used membrane mimics and the resulting collective membrane properties (13–15). At the same time, several studies also indicate that AMPs may have intracellular targets (16, 17), which might be coupled to highly specific interactions with cytosolic components and lead to the inhibition of different metabolic or biosynthesis pathways. Such multiple inhibitory processes, however, do not exclude any AMP activity within the bacterial membranes (17).

In order to shed some light on this issue, it would be highly instructive to compare AMP partitioning studies in microbes and artificial lipid-only membranes. However, and to the best of our knowledge, such data are currently not available. Instead, the peptide-to-lipid molar ratio commonly used in lipid-only systems, for example, dye leakage experiments, is about five orders of magnitude lower than in microbial killing assays (2, 18, 19). Estimates based on the surface/volume ratio of bacteria consequently indicate a large number of non-membrane bound peptides per cell (18). This may, however, be strongly biased by not considering cell-surface deformation, vesiculation processes, or extracellular peptide aggregation (16, 20). Reflecting on such controversies, Wimley and Hristova (2) formulated almost 10 years ago several unanswered questions relating to AMP activity studies in model membrane systems and microbes. Answering these questions is often challenged by the different experimental windows and sensitivities of the applied techniques (21). Here, focusing on the lactoferricin derivatives LF11-215 (H-FWRIRIRR-NH_2_) and LF11-324 (H-PFFWRIRIRR-NH_2_), we set out to address three of these questions: *(i) How are vesicle leakage and microbial killing correlated? (ii) Is membrane binding the sole basis for selectivity?* and *(iii) Is membrane translocation required for activity?*

Derived from human lactoferrin, the peptide lactoferricin and its 11 amino acids fragment LF11 are well-known for their affinity to lipid A and antimicrobial activity (22), with LF11-215 and LF11-324 being among the most promising LF11 derivatives with significantly improved activities against a broad range of Gram-positive and Gram-negative strains (20, 23–25). LF11-215 leads, for example, to a damage of the cell wall of Gram-negative bacteria, including an increased distance between cytoplasmic and outer membranes, membrane ruffling and formation of external blebs (20, 25). The somewhat increased hydrophobicity of LF11-324 was linked to a stronger efficacy in planktonic cultures, but at the same time led to a lower cell specificity (25). Interestingly, LF11-215 was instead reported to be more effective against bacterial biofilms (26), which is probably related to its lower hydrophobicity, facilitating the peptide diffusion within the matrix of microorganisms. Model membrane studies, using selected bacterial lipid species, suggest that both peptides interact preferentially with the negatively charged cardiolipin (CL), inducing a segregation into peptide-enriched and peptide-poor lipid domains (25). Further, LF11-215 and LF11-324 were shown to efficiently kill *Escherichia coli*, but exhibited at the same time only moderate membrane permeabilization in lipid vesicles consisting of *E. coli* total lipid extracts even at high peptide-to-lipid ratios, while N-acylated LF11 derivatives showed high activity both against bacteria and lipid-only membranes (20). Further, also effects of LF11-215 and LF11-324 on membranes, such as thinning or stored curvature stress, were rather modest (20). This suggests that the activity of both peptides in cytoplasmic membranes might not be the primary cause for their high efficiency in killing Gram-negative bacteria.

In order to gain some deeper insight in view of the three questions quoted above, we therefore correlated the partitioning of peptides in both bacterial cells and lipid-only systems with their effects on bacterial growth inhibition, membrane permeabilization, and structure, focusing on *E. coli* as representative strain of Gram-negative bacteria to demonstrate the feasibility of our approach. In particular, we exploited a thermodynamic framework that allowed us to relate the total number of AMPs located within bacteria at the minimum inhibitory concentration (MIC) to the membrane-bound fraction, combining a standard microbiological assay with ζ-potential measurements. The same theoretical framework was also applied to different lipid-only mimics of bacterial cytoplasmic membranes to determine the partitioning of the peptides and their membrane associated effects as a function of lipid composition, combining Trp fluorescence and dye leakage assays with small-angle X-ray scattering (SAXS). In agreement with previous studies, we find that LF11-324 is more active against *E. coli* than LF11-215 (20, 25). Performing our studies as a function of cell density, it has been further demonstrated that this difference in activity is enhanced at elevated cell concentrations, but also that the number of cell-associated AMPs at the MIC is about 4 times lower for LF11-324, while the number of surface-bound AMPs is about equal within the error of our experiments. At the same time, however, our results provide evidence that the vast majority of both peptides (up to 95−99%) are located in intracellular compartments. A slightly (~ 1.3 times) higher membrane partitioning of LF11-215 was observed in lipid mixtures of palmityol-oleoyl-phosphatidylethanolamine (POPE), palmitoyl-oleoyl-phosphatidylglycerol (POPG), and tetra-oleoyl-cardiolipid (TOCL), which most closely mimic *E. coli* cytoplasmic membrane, while differences in peptide partitioning into POPE/POPG membrane mimics were negligible. The overall partition process of AMPs into these systems plateaued after 10−20 min. Pure POPG bilayers instead showed instantaneous uptake of the peptides, but disparate partitioning for LF11-215 and LF11-324. Dye leakage experiments in turn showed intriguing effects. While pure POPG membranes followed typical permeabilization behavior [see, e.g., (27)] and showed the highest peptide partitioning coefficient, POPE/POPG and POPE/POPG/TOCL vesicles exhibited a maximum leakage at low peptide concentration, followed by a decrease of permeabilization upon further addition of peptide. Using dynamic light scattering (DLS) and SAXS, we were able to attribute this effect to the formation of large aggregate structures containing collapsed lipid membranes, sandwiching the peptides, and capable of encapsulating the dyes. No aggregates with collapsed lipid bilayers were formed for POPG. While the initial formation of the collapsed bilayers was most rapid for TOCL-containing membranes (within 30 s), the overall process including a relaxation was slower and occurred over the course of ~ 2 h.

## 2. Materials and Methods

### 2.1. Samples

#### 2.1.1. Lipids, Peptides, and Chemicals

POPE, POPG, and TOCL were purchased from Avanti Polar Lipids (Alabaster, AL) as powder (purity >99%), and freeze-dried LF11-215 and LF11-324 (purity >95%) were obtained from PolyPeptide Laboratories (San Diego, CA). ANTS (8-aminonaphthalene-1,3,6-trisulfonic acid, disodium salt) and DPX (*p*-xylene-bis-pyridinium bromide) were purchased from Molecular Probes (Eugene, OR) and dimethyl sulfoxide (DMSO) from Sigma-Aldrich (Vienna, Austria). HEPES (purity >99.5%) and LB-agar and LB-medium (Luria/Miller) powders were purchased from Carl Roth (Karlsruhe, Germany). All other chemicals were obtained from Sigma-Aldrich (Vienna, Austria) in *pro analysis* quality.

#### 2.1.2. Bacterial Suspensions

Colonies of *E. coli* K12 5K (courtesy of Günther Koraimann, University of Graz) were grown in LB-agar plates at 37°C. Overnight cultures (ONCs) were prepared by inoculating a single colony in 3 ml LB-medium in sterile polypropylene conical tubes (15 ml), enabling bacterial growth under aerobic conditions for 12−16 h in a shaking incubator at 37°C. Main cultures were prepared by suspending an aliquot of the ONCs in 10 ml LB-medium in sterile polypropylene conical tubes (50 ml) and harvested in the middle of the exponential growth phase. Bacteria were then immediately washed twice and re-suspended in nutrient-free and isotonic phosphate-buffered saline (PBS) solution (phosphate buffer 20 mM, NaCl 130 mM) at pH 7.4. Bacterial concentration was measured via turbidity measurements. The optical density at λ = 600 nm (OD_600_) was acquired with the spectrophotometer Thermo Spectronic Genesys 20 (Thermo Fisher Scientific, Waltham, MA), where OD_600_ = 1 corresponds approximately to 8 ×10^8^ CFU/ml (where CFU is colony forming units).

#### 2.1.3. Lipid Vesicles

Lipid stock solutions for sample preparation were prepared in organic solvent chloroform/methanol (9:1, vol/vol) and phosphate assayed for quantification of lipid content (28). Lipid thin films for SAXS measurements were prepared by mixing appropriate amounts of lipid stock solutions to obtain samples composed of POPE/POPG (3:1, mol/mol) and POPE/POPG/TOCL (82:6:12, mol/mol) followed by solvent evaporation under a nitrogen stream at 35°C and overnight storage in a vacuum chamber. Dry lipid films were hydrated in HEPES-buffered saline (HBS) solution (10 mM HEPES, 140 mM NaCl, pH 7.4).

All hydrated samples were equilibrated for 1 h at 40°C followed by 5 freeze-and-thaw cycles using liquid N_2_ and intermittent vortex-mixing. Large unilamellar vesicles (LUVs) were obtained by 31 extrusions with a handheld mini extruder (Avanti Polar Lipids, Alabaster, AL) using a 100 nm pore diameter polycarbonate filter. Vesicle size and polydispersity were determined via DLS using a Zetasizer NANO ZSP (Malvern Panalytical, Malvern, UK). ANTS/DPX-containing vesicles were separated from free ANTS/DPX by exclusion chromatography using a column filled with SephadexTM G-75 (Amersham Biosciences, Little Chalfont, UK) fine gel swollen in iso-osmotic HBS. Phospholipid concentrations for all samples were determined by phosphate analysis.

#### 2.1.4. Peptides

Peptide stock solutions of LF11-215 and LF11-324 were prepared in PBS solution for bacteria and HBS solution for lipid vesicles. Due to the weak solubility of LF11-324 in buffer, AMP stock solutions were prepared by adding acetic acid and DMSO, up to 0.3% and 3% vol/vol, respectively. Prior to each measurement, the concentrations of such compounds were systematically lowered down to 0.01% acetic acid and 0.1% vol/vol DMSO (final pH 7.2), in order to minimize, or even remove, any effect on the membrane structure (29). AMP concentrations of the stock solutions were determined by comparing against the absorption band of the aromatic amino acids with the spectrophotometer NanoDrop ND-1000 (Thermo Fisher Scientific, Waltham, MA). Peptide stock solutions were stored in silanized glass tubes until use.

### 2.2. Methods

#### 2.2.1. Microdilution Assay

The antimicrobial activity of the AMPs on *E. coli* was tested using a modified susceptibility microdilution assay (30) in the bacterial concentration range of 5 × 10^5^ to 10^9^ CFU/ml. Bacterial suspensions were incubated at a given AMP concentration for 2 h at 37°C (control samples were incubated in buffer only). Then, cell growth was monitored upon addition of double concentrated LB-medium for about 20 h using a Bioscreen C MBR (Oy Growth Curves Ab, Helsinki, Finland).

Data were analyzed assuming that the AMP-induced delayed bacterial growth is entirely due to a lower number density of survived cells, which is supported by the observation that the growth rate in the exponential phase does not depend on peptide concentration (Supplementary Figure 1A), i.e., the growth of the viable fraction of cells is similar to bacterial growth in absence of AMPs. This allowed us to quantify the number density of surviving bacteria from the onset of the exponential growth via the relation Δ*t*_exp_ = *a* + *b*log(*n*_cell_), where *a* and *b* were obtained from a calibration curve in absence of AMPs (Supplementary Figure 1B). Then, the inhibited bacterial fraction $\text{IF}=1-n_{\text{cell}}\left(\left[P\right]\right)/n_{\text{cell}}^{0}$ was calculated for different values of peptide concentration, [*P*], and of bacterial number density of the control sample, $n_{\text{cell}}^{0}$. Following the so-called “equi-activity” analysis (27, 31), we further interpolated IF using a Gompertz function, which enabled us to derive inhibitory peptide concentrations IC_*x*_, where *x* is the corresponding inhibited bacterial fraction (Supplementary Figure 1C). By convention, the standard MIC is defined at inhibition levels of 99.9%, i.e., IC_99.9_ ≡ MIC. Additional control experiments were performed in order to test whether DMSO and acetic acid, used in buffer solutions of both peptides (see above), affect bacterial growth. In particular, the microdilution assays were repeated with LF11-215 dissolved in pure PBS, yielding equivalently delayed onsets of bacterial growth. This suggests that DMSO and acetic acid at low concentrations used in this study do not significantly affect our derived MIC and IC_*x*_-values.

#### 2.2.2. Size and ζ-Potential Measurements

DLS and ζ-potential measurements were carried out with a Zetasizer Nano ZSP (Malvern Panalytical, Malvern, UK). *E. coli* suspensions were incubated with a given AMP concentration at 37°C for 1 h prior to each measurement. The used AMP concentrations were centered at the MIC values, and ranged from about 0.2 × to 2.5 × MIC. Control samples (no AMPs) were suspended and incubated in buffer alone. A concentration of 10^7^ CFU/ml was found to provide the optimal compromise between high signal-to-noise ratio and low multiple-scattering bias. Because of the high conductivity of PBS, the electrode voltage was set to 4 V for ζ-potential measurements to keep currents below 1 mA. Further, measurements were paused for 180 s between individual runs. This prevented Joule heating leading to sample denaturation and electrode blackening. Experiments were repeated three times on different preparations, each consisting of at least six measurements. Reported ζ-potential values are given by the median of the corresponding measurements (i.e., from at least 18 values) and errors were derived using the median absolute deviations.

#### 2.2.3. Leakage

Dye leakage experiments were performed combining previously reported protocols (20, 27). In brief, the dependencies of ANTS/DPX leakage on lipid and peptide concentration were determined by first incubating lipid vesicles ([*L*] = 1, 4, 10, and 20 mM) with peptides [[*P*] = (0.025−2) mM] at 37°C for 1 h using a gently rocking shaker (Eppendorf Thermomixer C, Hamburg, Germany). Similar time protocols were applied previously (27) and are justified here based on separate time-resolved leakage experiments (Supplementary Figure 3) and Trp-fluorescence measurements (Figure 3), showing that a quasi steady state has been reached after 1 h. Before measurements, lipid/peptide solutions were diluted with HBS to a lipid concentration of 50 μM, and every measurement was repeated at least twice. Vesicle size was checked after each incubation period using DLS. Samples were excited at λ= 360 nm, and the intensity of the fluorescence emission peak at λ= 530 nm, *I*_*p*_, was recorded with a slit width of 10 nm for both excitation and emission monochromators. Measurements were performed in quartz cuvettes in 2 ml of the iso-osmotic buffer on a Cary Eclipse Fluorescence Spectrophotometer (Varian/Agilent Technologies, Palo Alto, CA). The percentage of leakage, *E*_%_, was calculated according to the relation


(1) $$\begin{array}{l} E_{\%}=\frac{I_{p}-I_{min}}{I_{max}-I_{min}}, \end{array}$$

where *I*_*min*_ is the initial fluorescence without peptide, and *I*_*max*_ is the fluorescence corresponding to 100% leakage determined through the addition of a 1 vol% solution of Triton X-100. The initial, monotonic increase of *E*_%_ values with increasing [P] was interpolated with a sigmoidal function. This enabled us to obtain peptide and lipid concentrations leading to a specific *E*_%_ value, which can in turn be used to calculate the partitioning parameters (see section 2.3.1).

#### 2.2.4. Tryptophan Fluorescence

Fluorescence emission from Trp, present in both peptides, was measured with the Cary Eclipse Fluorescence Spectrophotometer, setting the excitation wavelength to λ= 280 nm, which corresponds to the maximum intensity of the Trp absorption/excitation band. Intensities of exciting and emitting light were adjusted by setting the slit widths of both incident and outgoing beam to 5 or 10 nm, depending on the emission intensity, in order to optimize the signal-to-noise ratio. Emission spectra were background-subtracted to remove contributions originating from the instrument's baseline and scattered light from vesicles. All samples were measured in HBS at 37°C using a quartz cuvette, with a magnetic stirrer to prevent sample sedimentation in the case of aggregation. LUVs ([*L*] = 100 μM) were mixed with peptides ([*P*] = 2 and 4 μM), recording fluorescence spectra at various post-mixing time intervals ranging from 0.5 to 60 min. Peptide solutions were measured at concentrations of 2, 3, and 4 μM in order to calibrate their intensity dependence in buffer.

The fluorescence emission band was fitted with the log-normal-like function (32, 33)


(2) $$\begin{array}{l} I(I_{0},\lambda ,\Gamma )=\\ \{\begin{array}{l} I_{0}\exp\left[-\frac{\ln2}{\ln^{2}\alpha }\ln^{2}\left(1+\frac{\left(\lambda -\lambda_{\text{max}}\right)}{y\Gamma }\right)\right],\;\lambda >\left(\lambda_{\text{max}}-y\Gamma \right)\\ 0,\;\lambda \leq \left(\lambda_{\text{max}}-y\Gamma \right) \end{array} \end{array}$$

where λ_max_ and *I*_0_ are, respectively, wavelength and intensity of the emission peak; Γ is the full-width-at-half-maximum (FWHM) of the band; α is a skewness parameter (fixed at an optimum values 1.36 after testing); and *y* = α/(α^2^ − 1). Spectra from mixtures of LUVs and peptides were analyzed with a linear combination of two independent bands *I*^*W*^ and *I*^*B*^, referring to AMPs in bulk (W) and partitioned into the lipid bilayer (B). λ^*W*^ and Γ^*W*^ were fixed to the reference values obtained by analyzing spectra from pure AMPs. Instead, $I_{0}^{W}$, $I_{0}^{B}$, λ^*B*^, and Γ^*B*^ were adjustable parameters. The so obtained set of $I_{0}^{W}$ values was converted to the concentration of dissociated peptides [*P*]_*W*_ and further analyzed for peptide partitioning as detailed in section 2.3.1.

#### 2.2.5. Small-Angle X-ray Scattering (SAXS)

SAXS experiments were performed at the highflux Austrian beamline at Elettra Synchrotron in Trieste, Italy (34) using a photon energy of 8 keV. SAXS patterns were recorded using a Pilatus 1 M detector (Dectris, Baden-Daettwil, Switzerland) in the *q*-range from 0.1 to 5 nm^−1^, and further processed with FIT2D (35). A custom-build cell, termed “nanodrop,” was used, allowing for precise measurements of very small volumes (10 μl) (36). Measurements were performed at a lipid concentration of 20 mg/ml (24.5−27.9 mM depending on the used membrane mimic) at 37°C. Lipids and peptides were mixed using an automatic sample changer and automatically injected into the nanodrop cell immediately after mixing. Peptide kinetics were measured starting 30 s after lipid-peptide mixing with an acquisition time of 1 s and a rest time of 10 s.

For the end-states, lipids mixed with peptides were incubated at 37°C for at least 4 h. These samples were measured using 12 frames of 10 s exposure each and a rest time of 12 s. Data were analyzed based on Bragg peak positions only. Using Bragg's law, the reported *d*-spacing values are simply given by *d* = 2π/*q*_*h*_, where *q*_*h*_ is the peak position.

### 2.3. Data Analysis

#### 2.3.1. Partitioning Equations for Lipid and Bacterial Systems

Following a previously reported thermodynamic formalism for the partitioning of peptides into lipid bilayers (37), based on the free energy of transfer of molecules from water into octanol (38), we define the mole fraction partitioning coefficient, *K*, as


(3) $$\begin{array}{l} K=\frac{{\left[P\right]}_{B}/\left({\left[P\right]}_{B}+\left[L\right]\right)}{{\left[P\right]}_{W}/\left({\left[P\right]}_{W}+\left[W\right]\right)}≃\frac{{\left[P\right]}_{B}\left[W\right]}{{\left[P\right]}_{W}\left[L\right]}, \end{array}$$

where [*P*]_*B*_ is the molar concentration of peptides partitioned into the lipid phase; [*P*]_*W*_ is the peptide concentration in the water phase; and [*L*] and [*W*] are, respectively, the molar concentrations of lipids and bulk water (55.5 M at 25°C and 55.3 M at 37°C). The approximation for *K* (Equation 3) is obtained applying [*W*] ≫ [*P*_*W*_] and [*L*] ≫ [*P*_*B*_] (similarly, the concentration of ions in water, i.e., buffer solutions, is negligible compared to [*W*]). By definition, the total concentration of peptides is [*P*] = [*P*]_*W*_ + [*P*]_*B*_, thus leading to


(4) $$\begin{array}{l} \left[P\right]=\underset{{\left[P\right]}_{W}}{\underset{︸}{\frac{R_{B}\left[W\right]}{K}}}+\underset{{\left[P\right]}_{B}}{\underset{︸}{R_{B}\left[L\right]}}=R_{B}\left(\frac{\left[W\right]}{K}+\left[L\right]\right), \end{array}$$

where *R*_*B*_ = [*P*]_*B*_/[*L*].

An analogous approach can be applied to cells (39), replacing the lipid membrane with a so-called “cell phase,” i.e., treating bacteria as a homogeneous medium consisting of all cell compartments accessible to the peptides. Then, similarly to Equation (3), the mole fraction partitioning coefficient, *K*_*C*_, of peptides in bacterial cells is defined as


(5) $$\begin{array}{l} K_{C}≃\frac{{\left[P\right]}_{B}\left[W\right]}{{\left[P\right]}_{W}\left[X\right]}, \end{array}$$

where [*X*] is the total molar concentrations of all molecular species within the cell phase, including cytoplasmic water. This leads to


(6) $$\begin{array}{l} \left[P\right]={\left[P\right]}_{B}\left(1+\frac{\left[W\right]}{K_{C}\left[X\right]}\right). \end{array}$$

Furthermore, [*P*]_*B*_ and [*X*] can be expressed as a function of the cell number density *n*_cell_:


(7) $${\left[P\right]}_{B}=\frac{N_{B}\;n_{\text{cell}}}{N_{A}}\text{ and }[X]=\frac{N_{X}\;n_{\text{cell}}}{N_{A}},$$

where *N*_*B*_ is the absolute number of partitioned peptides per single cell, *N*_*A*_ is Avogadro's constant, and *N*_*X*_, in analogy to [*X*], represents the *number of molecules that constitute the accessible compartments of a single cell*. The combination of Equations (6) and (7) gives:


(8) $$\begin{array}{l} \left[P\right]\left(n_{\text{cell}}\right)=\underset{{\left[P\right]}_{W}}{\underset{︸}{\frac{N_{B}\left[W\right]}{N_{X}K_{C}}}}+\underset{{\left[P\right]}_{B}}{\underset{︸}{\frac{N_{B}}{N_{A}}n_{\text{cell}}}}=\frac{N_{B}}{N_{A}}\left(\frac{N_{A}\left[W\right]}{N_{X}K_{C}}+n_{\text{cell}}\right) \end{array}$$

Since *N*_*X*_ is inaccessible, we thus define the *effective partitioning coefficient* $K^{eff}=N_{X}K_{C}$ as measurable quantity.

#### 2.3.2. Estimating the Maximum Number of AMPs Adsorbed to the Bacterial Surface

Remembering that ζ-potential values are sensitive only to the charges exposed to the outside of any particle, including bacteria, we can obtain upper boundaries for the number of surface adsorbed peptides. Given the high abundance of lipopolysaccharides (LPS) in the outer leaflet (about 70–90 vol%) of Gram-negative bacteria, as compared to other charged lipid species such as PG and CL (<2 vol%) and membrane proteins (7–20 vol%) (40), it is justified to assume that LPS dominates the surface potential of *E. coli*. Then, following considerations put forth for peptides partitioning in liposomes (31, 41), the ratio of ζ-potential values in the presence and absence of AMPs is


(9) $$\frac{\zeta }{\zeta_{0}}\approx \frac{\sigma }{\sigma_{0}}≃\left(\frac{S_{0}}{S}\right)\frac{\sum_{i}N_{i}z_{i}+N_{P}z_{P}}{N_{\text{LPS}}^{0}z_{\text{LPS}}},$$

where (σ, σ_0_) and (*S*, *S*_0_) are the corresponding surface charge densities and cell surface areas, respectively. Further, $N_{\text{LPS}}^{0}$ is the initial number of LPS molecules in the outer leaflet, with a nominal charge *z*_LPS_ ≃ −6 (42); *N*_*P*_ is the number of surface adsorbed AMPs of nominal charge *z*_*P*_ ≃ +5 (20); and $\sum_{i}N_{i}z_{i}=N_{\text{LPS}}z_{\text{LPS}}+N_{\text{PG}}z_{\text{PG}}+N_{\text{CL}}z_{\text{CL}}$ considers anionic lipids that translocate into the outer leaflet due to AMP activity (3, 6, 18). This term also accounts for a possible loss of LPS molecules in the outer leaflet due to vesiculation processes (25). Note that Equation (9) is only valid for |ζ| ≤ 25 mV, in which range ζ ∝ σ (43). Equation (9) also assumes that the position of the slipping plane is not significantly altered upon the addition of peptides. Rearranging Equation (9) leads to


(10) $$\begin{array}{l} \frac{N_{P}}{N_{\text{LPS}}^{0}}≃\left(\frac{z_{\text{LPS}}}{z_{P}}\right)\left(\frac{\zeta }{\zeta_{0}}\frac{S}{S_{0}}-\frac{\sum_{i}N_{i}z_{i}}{N_{\text{LPS}}^{0}z_{\text{LPS}}}\right), \end{array}$$

which is physically meaningful only if


(11) $$\begin{array}{l} \frac{\zeta }{\zeta_{0}}\frac{S}{S_{0}}\leq \frac{\sum_{i}N_{i}z_{i}}{N_{\text{LPS}}^{0}z_{\text{LPS}}}\leq 1. \end{array}$$

This leads to two extreme cases, the first one being the maximum “charge scrambling”


(12) $$\begin{array}{l} \frac{\zeta }{\zeta_{0}}\frac{S}{S_{0}}=\frac{\sum_{i}N_{i}z_{i}}{N_{\text{LPS}}^{0}z_{\text{LPS}}}, \end{array}$$

leading to $N_{P}/N_{\text{LPS}}^{0}=0$, i.e., complete dissociation of peptides from the bacteria, which is physically not realistic. The second limiting scenario is given by $\sum_{i}N_{i}z_{i}=N_{\text{LPS}}^{0}z_{\text{LPS}}$, i.e., bacteria retain their original surface exposure of LPS, which yields, upon insertion into Equation (10),


(13) $$\begin{array}{l} \frac{N_{P}^{\text{max}}}{N_{\text{LPS}}^{0}}\approx \left(\frac{z_{\text{LPS}}}{z_{P}}\right)\left(\frac{\zeta }{\zeta_{0}}-1\right), \end{array}$$

where $N_{P}^{\text{max}}$ is the upper boundary of surface adsorbed peptides. Moreover, this assumes *S*/*S*_0_ ~ 1, which may be contradictory to previously observed bacterial cell shrinking in the presence of AMPs [see, e.g., (44)]. However, it can be justified in view of our goal to obtain an estimate the upper boundary values for $N_{P}^{max}$. $N_{\text{LPS}}^{0}$ can be estimated using $N_{\text{LPS}}^{0}\approx 0.9S_{0}/A_{\text{LPS}}$, where *A*_LPS_ ≃ 1.6 nm^2^ (45, 46) is the lateral area per LPS molecule and *S*_0_ is supplied by size measurements. The prefactor originates from considering a maximum surface coverage of 90% by LPS molecules (40). For example, using DLS, we measured a hydrodynamic diameter of *E. coli* K12 2*R*_*H*_ = 980 ± 30 nm, from which we calculate $S_{0}≃4.5\times 10^{6}$ nm^2^, approximating the bacteria's shape with a cylinder of radius *r* and length *l* using $R_{H}^{2}≃r^{2}/2+l^{2}/12$ and *r* ~ 400 nm (47). This yields $N_{\text{LPS}}^{0}\approx 3\times 10^{6}$.

## 3. Results

### 3.1. Antimicrobial Activity and Partitioning in Bacterial Systems

#### 3.1.1. Efficacy and Partitioning

**Figure 1:**
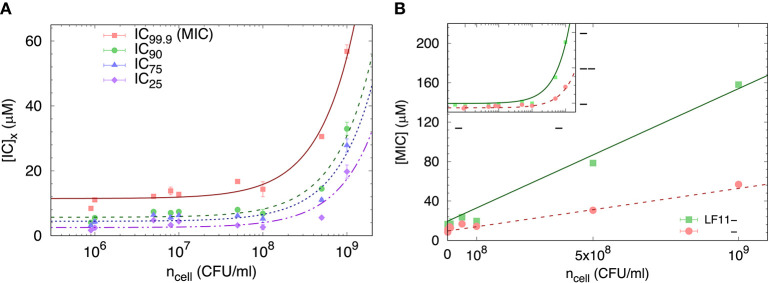
**(A)** Selected IC_*x*_-values of LF11-324 related to different inhibited fractions (see legend) as a function of *n*_cell_. Lines represent best fits using Equation (8). **(B)** Dependence of minimum inhibitory concentration (MIC) on the cell number density for LF11-215 and LF11-324, including best fits using Equation (8) (straight lines). The inset displays the same data on logarithmic scale.

We started our analysis of peptide activity in *E. coli* with measuring a range of inhibitory concentrations, including the MIC, by means of a slightly modified susceptibility assay, and for different bacterial concentrations. An example using LF11-324 is reported in Figure 1A, showing different IC_*x*_ values as a function of *n*_cell_ (note that in these bacterial suspensions 1 CFU corresponds to one single cell).

The partitioning formalism, described in section 2.3.1, was used to fit the linear increase of IC_*x*_ with cell concentration (Figure 1A) (LF11-215 data not shown). Also the MIC values increased linearly with cell concentration (Figure 1B), but more rapidly for LF11-215; results at *n* ≃ 5 × 10^5^ CFU/ml are consistent with previous reports (20, 25). At low concentrations, $n_{\text{cell}}\ll N_{A}\left[W\right]/K^{eff}\sim 2\times 10^{8}$ ml^−1^, [*P*] ≈ [*P*]_*W*_, explaining the apparent plateau in the semi-log plot at low bacterial densities (see inset in Figure 1B). In this regime, the number of peptides dispersed in buffer dominates the total AMP amount. At high concentrations, instead, i.e., $n_{\text{cell}}\gg N_{A}\left[W\right]/K^{eff}$, most of AMPs partition into the cell volume; thus [*P*] ≈ [*P*]_*B*_.

**Figure 2:**
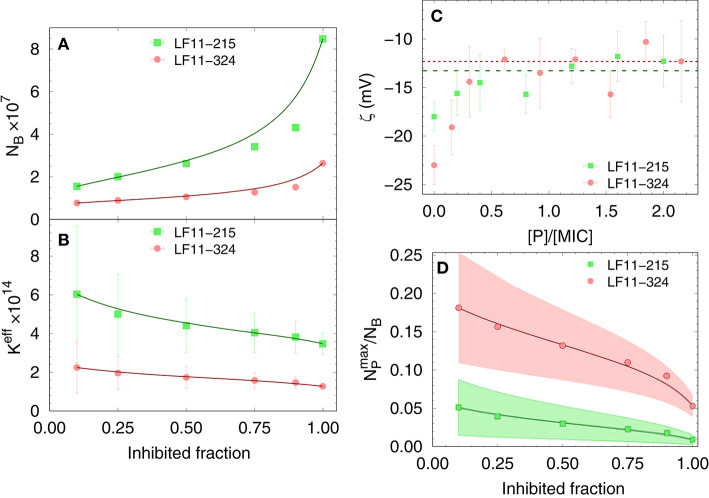
Dependence of the total number of antimicrobial peptides (AMPs) partitioned into *E. coli* **(A)**, as well as the effective partitioning coefficient **(B)** on the inhibited bacterial fraction; lines are a guide to the eyes. **(C)** Variation of ζ-potential with peptide concentration [normalized by the respective minimum inhibitory concentrations (MICs)]. The lines mark the average, constant ζ-values from [*P*] = 0.3 × MIC to 2.5 × MIC for LF11-215 (green dashed line) and LF11-324 (red dotted line). **(D)** Upper boundaries of the ratio of surface-adsorbed to cell-partitioned AMPs as a function of inhibited fraction. Color-shaded areas represent confidence intervals; lines are guides to the eyes.

Finally, results for *N*_*B*_ and *K*^*eff*^ from our thermodynamic analysis are shown in Figures 2A,B. About four times less LF11-324 is needed to bind to the cells compared to LF11-215 in order to induce full growth inhibition (Figure 2A). Interestingly, this is not related to the affinity of the AMPs to the cell (see Figure 2B), as LF11-215 exhibits ~2-fold higher *K*^*eff*^ values over the whole range of inhibited fractions. Further, *K*^*eff*^ slightly decreases as a function of inhibited fraction for both AMPs, suggesting that the likelihood of interactions with cells becomes lower the more AMPs are added to the system.

#### 3.1.2. Outer Leaflet Distribution of AMPs

The distribution of charged AMPs on the outer leaflet of the outer cell membrane was interrogated by analyzing ζ-potential data. Unfortunately, the “equi-activity” approach (31) was not applicable, because ζ-potential differences from the control systems were weak in magnitude and constant over a wide range of [*P*] around the MIC values (Figure 2C). Nevertheless, the maximum number of peptides partitioned in the outer bacterial surface, $N_{P}^{\text{max}}$, can be estimated to be in the order of 10^6^ AMPs for both LF11 molecules. $N_{P}^{\text{max}}$ (corresponding to about one peptide over 2–3 LPS molecules) is rather constant in the concentration range of 0.3 × to 2.5 × [MIC], suggesting that the cellular surface is already saturated with peptides in the sub-MIC range. In addition, $N_{P}^{\text{max}}$ can be compared with the total number of peptides per cell, *N*_*B*_. The ratio $N_{P}^{\text{max}}/N_{B}$ as a function of the inhibited fraction, and hence of the peptide concentration, is displayed in Figure 2D. We found $N_{P}^{\text{max}}/N_{B}\leq$ 25% at the lowest measured inhibited fraction, followed by a decrease of about 1 and 5% at the MIC of LF11-215 and LF11-324, respectively.

### 3.2. Effects in Cytoplasmic Membrane Mimics

In order to compare the growth inhibition of *E. coli* by LF11-215 and LF11-324 with their membrane activity, we prepared 100 nm sized LUVs of three different cytoplasmic membrane mimics, pure POPG, POPE/POPG (3:1 mol/mol), and POPE/POPG/TOCL/ (82:6:12 mol/mol/mol), all of which are frequently used in biophysical studies on the mode of action of AMPs [see e.g., (15, 48–50)]. Out of these systems, POPE/POPG/TOCL/ (82:6:12 mol/mol/mol) most closely resembles the natural lipid composition of *E. coli* inner membranes (51).

#### 3.2.1. Tryptophan Fluorescence

**Figure 3:**
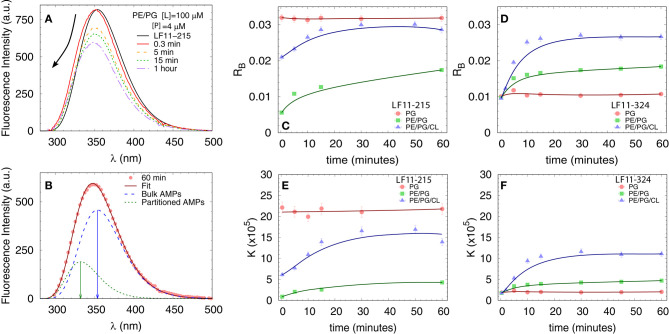
Peptide partitioning from Trp fluorescence experiments. **(A)** Example of Trp emission-band kinetics. The arrow indicates the decrease in intensity and blue-shift as a function of time. **(B)** Example of spectral analysis (POPE/POPG [*L*] = 100 μM, LF11-215 [*P*] = 4 μM); red line: best fit; blue dashed line: emission from antimicrobial peptides (AMPs) in suspension; green dotted line: emission from AMPs partitioned into the membrane. The arrows mark the different λ_max_ values. **(C,D)** Adsorption kinetics for LF11-215 and LF11-324, respectively, for three different membrane mimics. **(E,F)** Kinetics of the partitioning coefficient *K* for LF11-215 and LF11-324, respectively, for three different membrane mimics.

We first measured the partitioning of the two peptides using Trp fluorescence. The emission spectra of the single Trp residue of LF11-215 and LF11-324 exhibited in buffer a band with λ^*W*^ ≃ 354 nm and Γ^*W*^ ≃ 65 nm [see e.g., Figure 3A]. This is in agreement with the emission maximum of Trp exposed to a polar environment (52). When peptides were added to the LUVs, the Trp emission bands exhibited a blue-shift regardless of lipid composition and peptide type (example in Figure 3A). The analysis of the spectra (see example in Figure 3B and details in section 2.2.4) enabled us to derive the kinetics of AMPs partitioning into the different bilayers. For all membranes, the fluorescence signal from the partitioned AMPs exhibited values of λ^*B*^ ≃ (331 − 335) nm and Γ^*B*^ ≃ (49 − 54) nm, indicating an average location of the Trp residues within the hydrophobic region of the lipid bilayer (52). CL-containing systems displayed broader bands, Γ^*B*^ ≈ 65 nm, after 20−30 min of incubation, probably due to a heterogeneous distribution of Trp locations within the bilayers (33). Speculating that this might be related to supramolecular structural changes, we performed DLS after 60 min of incubation. Indeed, we observed large aggregate structures, for POPE/POPG/TOCL samples, but also for POPE/POPG in presence of 4 μM LF11-324 (see Supplementary Figure 2A and Table 1). We note, however, that the derived AMP partitioning data are not directly linked to such morphological changes. Overall, CL and LF11-324-containing samples showed the highest propensity to form aggregates, whereas POPG LUVs remained intact under present conditions.

**Table 1:** Partitioning parameters calculated from the spectral analysis of the Trp emission band for LF11-215 and LF11-324.

**LF11-215 (2 μM / 4 μM)**	***R*_*B*_** **×10^−3^**	**K ×10^4^**	**[*P*]_*W*_ (μM)**
POPG	14.9 ± 0.3 / 31.9 ± 0.4	161 ± 6 / 217 ± 8	0.513 ± 0.015 / 0.81 ± 0.04
POPE/POPG	11.2 ± 0.3 / 17.5 ± 0.7	70 ± 4 / 43 ± 3	0.88 ± 0.03 / 2.25 ± 0.07
POPE/POPG/TOCL^*^	11.9 ± 0.2 / 28.6 ± 0.3	82 ± 4 / 139 ± 6	0.81 ± 0.02 / 1.14 ± 0.03
**LF11-324 (2** **μM / 4** **μM)**	***R*_*B*_** **×10^−3^**	**K** **×10^4^**	**[*P*]_*W*_** **(μM)**
POPG	10.5 ± 0.3 / 10.7 ± 0.9	61 ± 4 / 20 ± 20	0.95 ± 0.03 / 2.93 ± 0.09
POPE/POPG^*^	11.0 ± 0.3 / 18.4 ± 0.6	67 ± 4 / 47 ± 3	0.90 ± 0.02 / 2.16 ± 0.06
POPE/POPG/TOCL^**^	10.6 ± 0.3 / 26.7 ± 0.4	63 ± 4 / 111 ± 5	0.95 ± 0.03 / 1.33 ± 0.04

*Data refer to systems after 1 h of incubation with peptides (see Figure 3), and [L] = 100 μM*.

* *Samples showing aggregation at [P] = 4 μM*;

** *Samples showing aggregation at [P] = 2 and 4 μM*.

Table 1 reports results of the AMP partitioning analysis (see section 2.1) after 60 min of incubation with the peptides, and the temporal evolution of these parameters for [*P*] = 4 μM is shown in Figures 3C–F. Both peptides quickly achieve stationary *R*_*B*_ and *K* values in POPG LUVs (within 30 s), but the amount of adsorbed LF11-215 is three times higher than for LF11-324. In PE-containing bilayers both peptides instead exhibited similar adsorption and partitioning kinetics, with an increase over the first 10-20 min before reaching stable values. Both peptides showed, however, a higher affinity to POPE/POPG/TOCL membranes than to POPE/POPG. In contrast, at lower peptide concentration (2 μM), this effect was not observed (Table 1). In this case, the parameters derived from the partitioning analysis including POPG did not depend on whether LF11-215 or LF11-324 was added.

#### 3.2.2. Leakage

**Figure 4:**
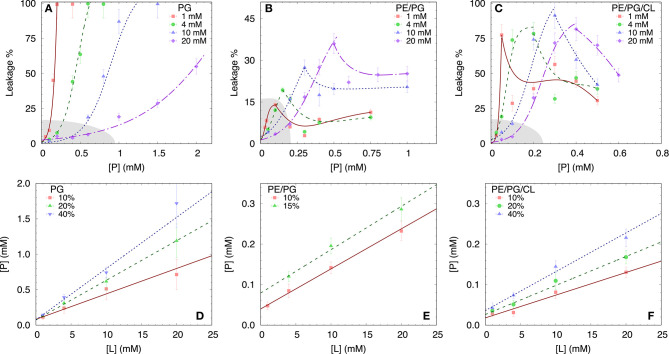
LF11-215 induced permeabilization of LUVs composed of POPG **(A)**, POPE/POPG **(B)**, and POPE/POPG/TOCL **(C)** at different lipid concentrations (see legend). A sigmoidal function was used to interpolate the initial leakage increase, whereas lines describing the following decrease **(B,C)** are just guides to the eyes. Gray-shaded areas indicate regimes where the formation of large aggregates was not detected. **(D–F)** Analysis of peptide induced ANTS/DPX leakage (see legend) in terms of Equation (4) (straight lines) for the different membrane mimics. For result, see Table 2.

To test the permeability of the three membrane mimics, we investigated the dye efflux from LUVs after incubation with LF11-215 at various lipid-to-peptide ratios. This allowed us to pursue the effect of peptides at a physiological temperature, where all lipid systems are in the fluid phase, up to very high lipid concentrations typically used in small angle scattering experiments ([*L*] ~20 mM). Figures 4A–C display leakage percentages as a function of [*P*] for different concentrations of POPG, POPE/POPG, and POPE/POPG/TOCL systems. In all three systems, substantial leakage seemed to coincide with the formation of larger structures, as corroborated with DLS measurements (see example in Supplementary Figure 2B). Indeed, the transformation from unilamellar vesicles to larger aggregates (marked as a gray, shaded areas in Figures 4A–C) depends on the overall lipid and peptide concentrations, and coincides with a leakage of 15−20%, and [*P*] ≤ 1 mM for POPG and [*P*] ≤ 0.25 mM for PE-containing systems. Interestingly, while POPG LUVs showed a sharp, sigmoidal increase of leakage up to 100%, PE-containing systems exhibited a maximum at a certain peptide-to-lipid ratio, followed by a leakage decrease and, most likely, a stabilization at higher [*P*] values. POPE/POPG vesicles, in particular, resulted in a relatively low total efflux ≤ 40%. In analogy with the equi-activity analysis used for the susceptibility assay, leakage curves where exploited to calculate the partitioning parameters (27). The peptide concentrations needed to induce leakage *E*_%_ at a given [*L*] were all fitted with Equation (4), demonstrating that in these ranges of [*P*] and [*L*], the partitioning characteristic are alike, regardless of transitions from a unilamellar system to more complex lipid aggregates. Fitting results are shown in Table 2. POPG samples seem to require a high number of LF11-215 per lipid in order to achieve leakage, showing the highest *R*_*B*_ and *K* values, which increase along with higher leakage, and with a rather constant [*P*]_*W*_. This means that at [*L*] ≃20 mM, > 90% of peptides are partitioned into the lipid phase. Leakage from PE-containing systems instead needs less AMPs per lipid (see Table 2), with a maximum amount of partitioned AMPs of <80% for [*L*] ≃20 mM.

**Table 2:** Partitioning parameters for LF11-215, resulting from the leakage assay analysis of three differently composed LUVs (see also Figure 4).

***Leakage***	***R*_*B*_** **×10^−3^**	**K ×10^4^**	**[*P*]**_***W***_ **(mM)**
POPG 10%	36 ± 4	2.4 ± 0.7	0.08 ± 0.03
POPG 20%	55.9 ± 1.6	4.2 ± 0.4	0.073 ± 0.009
POPG 40%	72 ± 6	5.4 ± 1.4	0.08 ± 0.03
POPE/POPG 10%	9.8 ± 0.4	1.37 ± 0.16	0.040 ± 0.006
POPE/POPG 15%	10.7 ± 1.0	0.75 ± 0.15	0.08 ± 0.02
POPE/POPG/TOCL 10%	5.6 ± 0.7	1.7 ± 0.8	0.018 ± 0.010
POPE/POPG/TOCL 20%	7.2 ± 0.5	1.5 ± 0.3	0.026 ± 0.007
POPE/POPG/TOCL 40%	9.6 ± 0.9	1.5 ± 0.4	0.036 ± 0.012

*Concentration ranges: [L] = (1−20) mM, [P] = (0.05−2) mM*.

#### 3.2.3. SAXS

**Figure 5:**
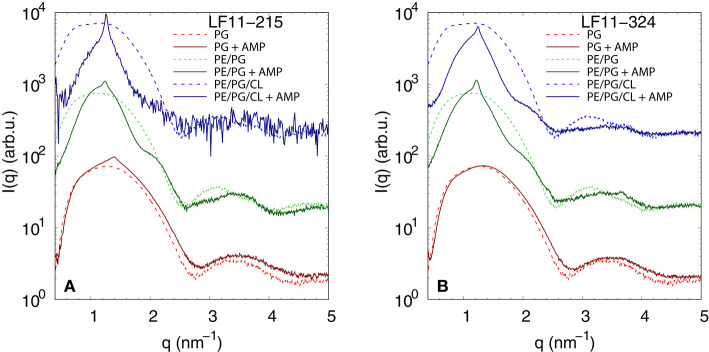
SAXS patterns of POPG, POPE/POPG, and POPE/POPG/TOCL before and after 4 h after incubation with **(A)** LF11-215 and **(B)** LF11-324 (end-states) at [*P*]/[*L*] = 1:25, corresponding to [*L*] = (24.5−27.9 mM) and [*P*] ~ 1.1 mM.

**Figure 6:**
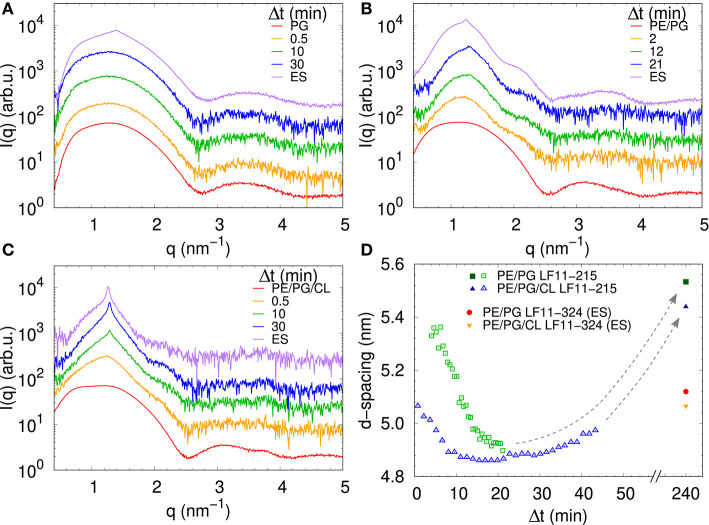
LF11-215-induced structural kinetics as observed in the evolution of SAXS patterns of **(A)** POPG, **(B)** POPE/POPG, and **(C)** POPE/POPG/TOCL; [*P*]/[*L*] = 1:25, corresponding to [*L*] = (24.5–27.9 mM) and [*P*] ~ 1.1 mM. Panel **(D)** shows the changes in *d*-spacing over time for POPE/POPG and POPE/POPG/TOCL with LF11-215, as well as end-states (ES) for LF11-215 and LF11-324 measured after 4 h of system equilibration.

We performed SAXS experiments to investigate structural changes in membrane mimics induced by the peptides. Vesicles composed of POPG, POPE/POPG, and POPE/POPG/TOCL without AMPs (reference systems) as well as end-states at a lipid-to-peptide ratio of 1:25 measured 4 h after lipid-peptide mixing are shown in Figures 5A,B for LF11-215 and LF11-324, respectively. All reference systems showed purely diffuse scattering patterns originating from positionally uncorrelated lipid bilayers, as expected for LUVs. In the case of POPG liposomes, after addition of either peptide, a shift of the first minimum at *q* ~ 2.8 nm^−1^ can be observed, which could originate from a thinning of the membrane. Most pronounced for PE-containing mimics, the measurements after peptide mixing showed a small, but clearly discernible positional correlation peak at *q* ~ 1.1 − 1.3 nm^−1^ in addition to a significant diffuse scattering pattern; a similar feature—but much less expressed—was also observed for POPG in the presence of LF11-215. The diffuse scattering of POPE/POPG and POPE/POPG/TOCL mixtures in the presence of both peptides showed an additional modulation at *q* ~ 2 nm^−1^. This feature might originate from a preferential enrichment of AMPs in one leaflet or the formation of thin peptide-enriched domains. Clarification of the underlying structures would require dedicated experiments using neutron scattering combined with contrast variation and computational modeling [see e.g., (49)]. This is, however, beyond the scope of the present study. Instead, we focused on the evolution of the correlation peak in case of LF11-215 using time-resolved SAXS (Figure 6). For the PE-containing samples, especially in the case of POPE/POPG/TOCL, addition of peptides led to a rapid precipitation of the sample, which reduced the amount of sample being hit by the X-ray beam. This explains the increased noise of scattering data at longer times. For liposomes consisting of pure POPG, only the final pattern contained a weak feature of a positional correlation peak. In turn, TOCL-containing membrane mimics showed the onset of peak formation already 30 s after mixing, while this was slightly delayed to about 5 min in POPE/POPG. The *d*-value, derived directly from the peak position, exhibited a non-monotonic behavior over time in both POPE/POPG and POPE/POPG/TOCL mimics (Figure 6D), showing first a decrease and subsequent increase over several minutes after peptide addition. During this equilibration process, the *d*-values of POPE/POPG were always larger than those of POPE/POPG/TOCL mixtures, with a difference of ~0.1 nm in the end-states. Interestingly, final *d*-values of both PE-containing mixtures were even lower in the presence of LF11-324 (POPE/POPG: *d* = 5.12 nm and POPE/POPG/TOCL: *d* = 5.07 nm). This could stem either from a pronounced membrane thinning or/and different penetration depths of the peptides within the bilayer (see section 4). Notably, the time scale of the related equilibration process is much longer (hours) than peptide adsorption (minutes) (see Figure 3).

## 4. Discussion

We exploited a common thermodynamic framework for the partitioning of antimicrobial peptides to bridge the activities of LF11-215 and LF11-324 in *E. coli* and different lipid-only mimics of their cytoplasmic membranes. In the case of bacteria, this was achieved by evolving a susceptibility microdilution assay for the antimicrobial activity of the two AMPs. This yielded more accurate results (confidence < 3%) than standard MIC evaluations over a broad range of cell concentrations. In particular, we found MIC_LF11-215_ = (13.7 ± 0.4) μM and MIC_LF11-324_ = (11.1 ± 0.6) μM at $n_{\text{cell}}=10^{6}$ CFU/ml [in agreement with (20, 25)] increasing linearly up to MIC_LF11-215_ = (158 ± 2) μM and MIC_LF11-324_ = (57 ± 2) μM at $n_{\text{cell}}=10^{9}$ CFU/ml. The higher antimicrobial efficacy of LF11-324 was conserved for all measured cell concentrations and extended also to lower growth-inhibited fractions at decreased AMP concentrations. Intriguingly, LF11-324 showed a lower partitioning coefficient than LF11-215, suggesting energetically less-favored interaction with cells, despite its higher efficacy. Combing these data with ζ-potential measurements further allowed us to obtain an upper boundary for the surface adsorbed AMP fraction. Strikingly, we found that both lactoferricin derivatives bind about equally to the microbial envelope, but constitute only a minor fraction of the total number of AMPs interacting with the bacteria. That is, at the MIC only 1−5% of AMPs are surface bound, and even at bacteria inhibition levels of 10% only ~ 5% (LF11-215) to ~ 18% (LF11-324) of the cell associated peptides do not enter inner compartments (Figure 2). Consequently, both peptides target mainly inner bacterial components.

Partitioning of LF11-215 and LF11-324 in lipid membrane mimics was instead investigated using Trp fluorescence and ANTS/DPX leakage. While the first technique quantifies the actual number of partitioned peptides per lipid, the second provides the number of adsorbed AMPs leading to a specific dye-efflux. Trp fluorescence revealed a faster and stronger partitioning into POPG membranes than into PE systems in the case of LF11-215, whereas LF11-324 showed the lowest adsorption and partitioning into POPG. Based on pure electrostatic interactions of anionic POPG and cationic residues, which are identical for both peptides, this appears counterintuitive. It can be understood, however, considering that the amphipathic moment of both LF11 peptides is normal to the membrane plane, with the aromatic amino acids of the N-terminal inserted into the hydrophobic core of the membrane, and the Arg-rich section of the amidated C-terminal interacting with the lipid head-groups and exposed to the solvent as reported from previous studies on this family of peptides (25, 53). Indeed, our fluorescence data show that the Trp side chain is segregated into the apolar environment of the bilayer, hence suggesting that the protonated amino group of the N-terminal lies in the same region. Consequently, the positively charged N-terminus of LF11-324 needs, because of the additional Phe and Pro residues, to insert deeper into the hydrophobic core of the bilayer and thus further away from the anionic PG than in the case of LF11-215, leading to an unfavorable configuration. In the case of PE-containing mixtures, such electrostatic interactions are less pronounced; moreover, peptide-induced domain formation, as previously observed for CL-containing mixtures in the presence of LF11 derivatives (20, 25), may facilitate peptide insertion due to packing defects at domain boundaries [see e.g., (54)]. Hence, POPE/POPG and POPE/POPG/TOCL mixtures are thus more reliable mimics of *E. coli* inner membranes than pure POPG. Moreover, the approximate similar adsorption of both peptides (Figures 3C,D) resembles roughly the findings in *E. coli* (Figure 2D), thus suggesting that these mimics are also first-order proxies to peptide partitioning into outer membranes, despite the lack of LPS.

Dye leakage experiments for PE-containing mimics showed an effect that, to the best of our knowledge, has not been reported before. Upon raising peptide concentration, we observed an initial increase of dye-efflux, peaking at relatively low peptide concentrations, followed by a subsequent decrease of permeabilization upon further addition of peptide (Figures 4A–C). POPG vesicles in contrast showed a typical pure sigmoidal increase of permeabilization [see e.g., (27)], but required significantly higher peptide concentrations than in other mixtures, which aligns with our above discussion on competing electrostatic interactions of the charged N-terminus with PG headgroups. Moreover, DLS experiments revealed that significant leakage was always associated with the formation of large aggregates, independent of the lipid membrane mimic (i.e., also for pure POPG). The intricate entrapment of dyes in POPE/POPG and POPE/POPG/TOCL membranes at increased levels of AMP concentration can be rationalized considering our SAXS data. Here all three lipid mixtures, with the exception of POPG in the presence of LF11-324, showed the formation of a weak Bragg peak (Figure 5), signifying the presence of a minor fraction of positionally well-correlated aggregates.

While a sole correlation peak does not enable a conclusion about the specific supramolecular structure, it is still possible to derive *d*-spacing values from the peak positions, which can be connected to previous studies on peptide-induced multibilayer systems with collapsed interbilayer distance (10). Consider first POPE/POPG (3:1 mol/mol) and its steric bilayer thickness, which has been previously determined with high accuracy to be ~ 4.7 nm (49). The final *d*-values for this system after addition of LF11-215 and LF11-324 were 5.53 and 5.12 nm. This suggests that the Bragg peaks originate from lamellar aggregates with almost completely collapsed bilayers, most likely only separated by the steric size of peptides partially inserted into the membranes. Thus, the lower *d*-value observed for LF11-324 suggests pronounced membrane thinning as a result of a stronger perturbation of the membrane, which could be due to the bigger size of the peptides' hydrophobic patch, leading to stronger disordering effects within the hydrocarbon chain region.

For TOCL containing mixtures, we found slightly lower final *d*-values for both peptides, but again more pronounced for LF11-324 (Figure 6D). Since we do not have any reference data on the steric thickness of POPE/POPG/TOCL (82:6:12 mol/mol/mol) bilayers, this might be due to either differences in membrane thickness in the absence of peptide or peptide-induced membrane perturbation. However, given that the hydrocarbon thickness of TOCL is expected to be about equal to POPG and POPE, we speculate that the smaller *d*-values for POPE/POPG/TOCL are due to an increased membrane perturbation by the peptides. Indeed, a more pronounced effect of peptides for TOCL containing mixtures, as compared to POPE/POPG, was also observed in Trp fluorescence experiments, where these mixtures exhibited the higher partitioning coefficients, as well as faster kinetics (Figure 3). Faster kinetics were also observed for the formation of the collapsed lamellar phase in the presence of LF11-215, where the weak Bragg peak was present in POPE/POPG/TOCL 30 s after the addition of peptides, but needed about 5 min to form in POPE/POPG (Figure 6D). The initial decrease in *d*-spacing observed for the first 10−20 min interestingly correlates with the Trp fluorescence kinetics. This suggests that the shift of the peak position over time is related to an initial accumulation of peptides on the membranes during approximately 20 min, followed by a slow equilibration over the course of the next few hours, where peptides diffuse into the newly formed aggregates, most of which are not forming collapsed multibilayers.

Interestingly, the lowest *d*-values of both membranes are ~ 4.9 nm (Figure 6D), which is about equal to the steric membrane thickness of POPE/POPG (49). This provides evidence that the membranes come in close contact. The negative intrinsic curvatures of POPE (55) and TOCL (56) and their well-known propensity to form non-lamellar structures [see e.g., (57)] make them highly prone to induce membrane fusion (58, 59). We thus propose that the *d* value minima indicate time points of membrane fusion. Note that peptide-induced fusion of membranes was reported previously (10). Membrane fusion would also explain why some of the dyes are not released by the peptides activity, but remain trapped within some aggregates (Figure 4). Interestingly, however, also POPG showed in the presence of LF11-215 a weak signature of collapsed bilayers after extended equilibration times (Figure 5A), with a *d* ~ 4.5 nm that is even lower than the smallest values observed for POPE/POPG and POPE/POPG/TOCL (Figure 5D). Note that POPG has an intrinsic curvature close to zero (15), and is well-known as a lamellar-phase forming lipid. The collapsed lamellar structure formed in POPG is thus unlikely to be related to membrane fusion, but merely the result of possibly disintegrated membrane patches, forming a stack of bilayers. This notion is supported by the high permeability of POPG vesicles and the absence of a drop of dye leakage at increased peptide concentrations (Figure 4A).

The overall higher peptide activity in PE and PE/CL-containing mixtures (as observed, for example, by the lower amounts of peptide needed to induce dye leakage) further suggests that the intrinsic lipid curvatures of these lipids and the resulting stored elastic energy stress (60) makes the bilayers more vulnerable to the peptides. A similar view has been previously proposed as the “balanced spring model for membrane interactions” (61), where membrane active compounds can relief the stored elastic stress in bilayers upon insertion. Finally, we note that also LPS is known for their propensity to form non-lamellar structures (62, 63). We thus expect analogous driving forces occurring in the bacterial outer membrane, although details of membrane composition and in particular asymmetry certainly cannot be neglected. *Nota bene*, we do not necessarily expect that both peptides induce membrane fusion in live bacterial systems, as the complex architecture of the cell envelope or the presence of a densely packed cytosol provides additional constrains. The occurrence of fusion events in lipid-only systems is a mere indication for elastic curvature stress stored within the membranes, which upon relaxation in the presence of peptides will assist their translocation into the cytosol.

In conclusion, our results show that bridging peptide activities in live bacteria and lipid membrane mimics is intricate and that a simple delineation of common leakage experiments can be highly misleading. In particular, for the here studied LF11-215 and LF11-324, one might have concluded based on leakage data that pure POPG would be sufficient to explain the membrane permeabilizing potential of both peptides. Yet, detailing the partitioning of the peptides in different membrane mimics and microbes allowed us to arrive at a completely different picture. Instead, LF11-215 and LF11-324 both target mainly intracellular components, presumably DNA due to its highly negative charge, reaching local concentrations of up to ~ 100 mM at the MIC. Moreover, the elastic curvature stress stored in POPE/POPG, and to an even greater extent in the more realistic POPE/POPG/TOCL mixture, bestows the membranes with an increased potential for peptide translocation, which might in general be exploited by surface adsorbed wedge-like AMPs. Thus, at least for the presently studied peptides we may answer the initially posed questions as follows: *(ad i)* Vesicle leakage and microbial killing are not directly correlated, but some valuable insight can be obtained upon coupling leakage data to peptide partitioning and studies of membrane structural changes. *(ad ii)* Both peptides bind about equally well to membranes, so for this part, their selectivity indeed seems determined by membrane binding. The difference in their efficacy, however, is due to specific interactions with cytosolic components, and most likely DNA. *(ad iii)* Our partitioning studies provide strong evidence that membrane translocation is definitely required for LF11-215 and LF11-324. Additional studies using different bacteria and AMPs need to be performed to verify whether it is possible to generalize these findings. In general, and although we restricted our study to two AMPs and a single bacterial strain, the developed framework provides a path and guidelines for future studies intending to get deep insight on AMP activity by coupling *in vitro* cell studies with lipid membrane mimics.

## Data Availability Statement

The original contributions presented in the study are included in the article/Supplementary Materials, further inquiries can be directed to the corresponding author/s.

## Author Contributions

LM, ES, NM, GP, and KL conceptualized and designed the study. LM, ES, JM, JK, MF, and NM carried out the experiments. LM, ES, JM, and GP carried out the data analysis and interpretation. LM, ES, and GP wrote the manuscript. All authors provided critical feedback and helped to shape the research, analysis, and manuscript.

## Conflict of Interest

The authors declare that the research was conducted in the absence of any commercial or financial relationships that could be construed as a potential conflict of interest.
