# Supplementary material for: Bridging the Antimicrobial Activity of Two Lactoferricin Derivatives in E. coli and Lipid-Only Membranes
Source: Front Med Technol. 2021 Feb 24;3:625975. doi: 10.3389/fmedt.2021.625975 (PMC8757871; doi:10.3389/fmedt.2021.625975)
Supplement: Supplementary file 1 [file Data_Sheet_1.pdf]

Supplementary Material

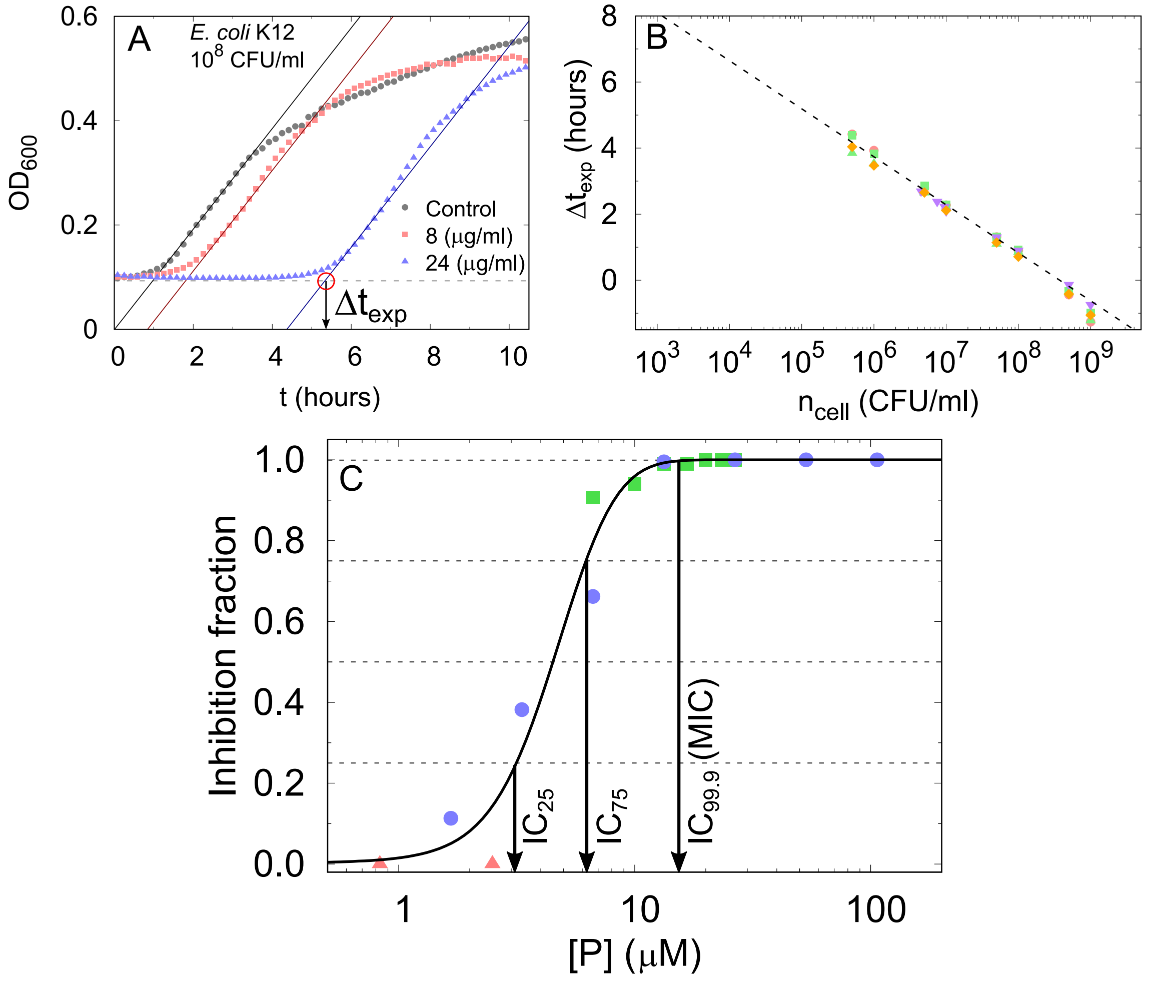

Figure S1. **(A)** Example of delayed growth as a function of AMP concentration, and extraction of $t_{\rm exp}$ values. **(B)** Calibration: logarithmic description of $t_{\rm exp}$ as a function of $n_{\rm cell}$. **(C)** Sigmoidal interpolation of the inhibition fraction data as a function of AMP concentration (different colors represent independent experiments). As an example, arrows highlight the calculated IC_x in the case of inhibition fraction 0.25, 0.5 and 0.999 (MIC).

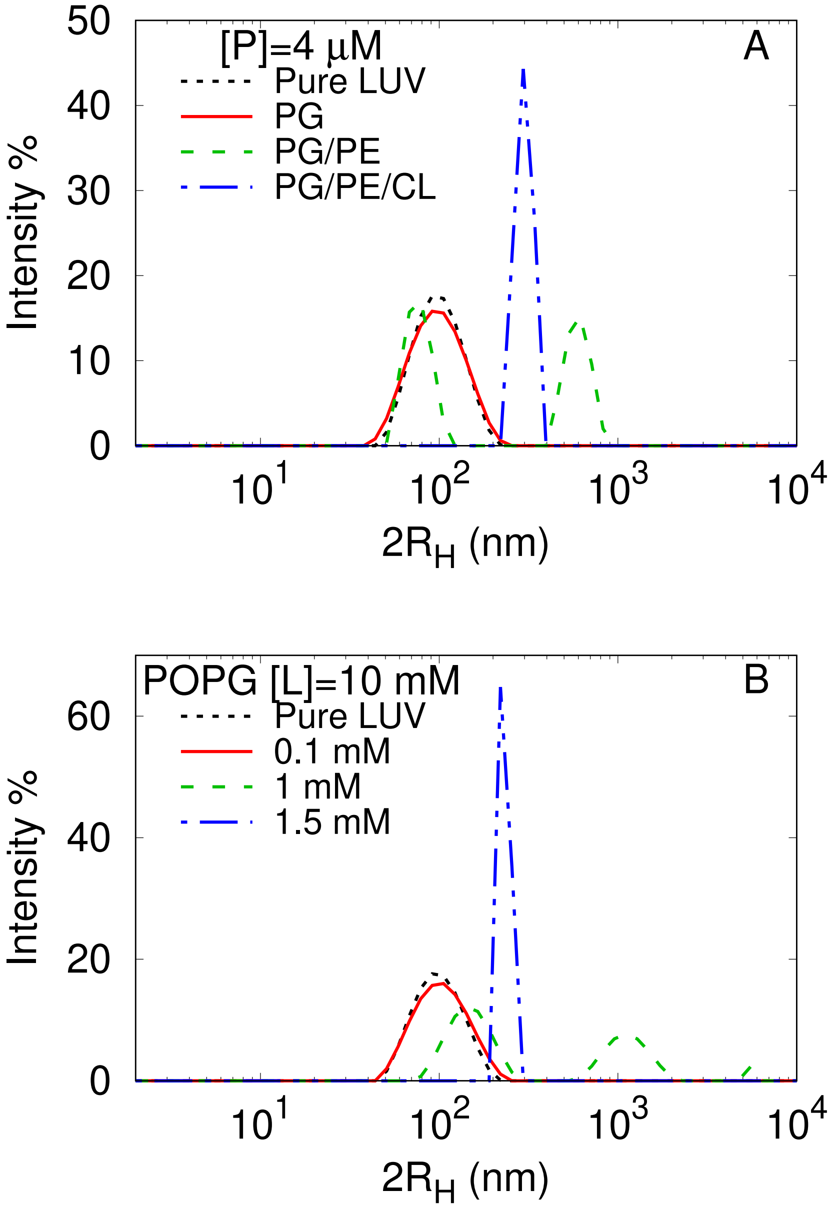

Figure S2. **(A-B)** Representative size distribution functions (intensity-weighed) after incubation with peptides for one hour at 37 °C. **(A)** Different lipid systems ($[L] = 100\ \mu$M) after incubation with LF11-215 ($[P] = 4\ \mu$M). **(B)** Different LF11-324 concentrations and mixing with POPG LUVs ($[L] = 10$ mM).

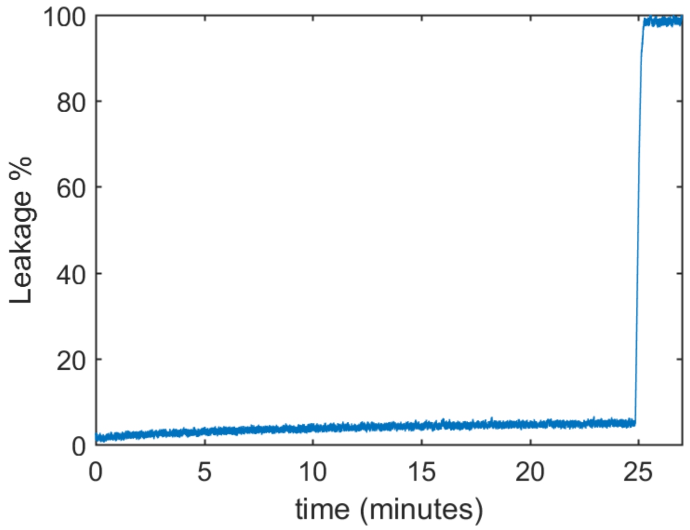

Figure S3. Time-resolved measurement of LF11-215($[P] = 0.5$ μM) induced permeabilization of LUVs composed of POPE/POPG ($[L] = 50$ μM), showing peptide induced ANTS/DPX leakage kinetics after peptide addition converging to $\sim$ 5 % dye efflux after 25 min. The jump to 100% leakage is induced by addition of Triton X-100.
